# Association between Floods and Acute Cardiovascular Diseases: A Population-Based Cohort Study Using a Geographic Information System Approach

**DOI:** 10.3390/ijerph13020168

**Published:** 2016-01-28

**Authors:** Alain Vanasse, Alan Cohen, Josiane Courteau, Patrick Bergeron, Roxanne Dault, Pierre Gosselin, Claudia Blais, Diane Bélanger, Louis Rochette, Fateh Chebana

**Affiliations:** 1 Department of Family Medicine and Urgent Medicine, Faculty of Medicine and Health Sciences, Université de Sherbrooke, 3001 12th Avenue North, Sherbrooke (Québec), QC J1H 5N4, Canada; Alan.Cohen@USherbrooke.ca (A.C.); Josiane.Courteau@USherbrooke.ca (J.C.); Roxanne.Dault2@USherbrooke.ca (R.D.); 2 Research center of the Centre Hospitalier Universitaire de Sherbrooke (CHUS), 12th Avenue North, Sherbrooke (Québec), QC J1H 5N4, Canada; 3 Department of Biological Sciences, Bishop’s University, 2600 College Street, Sherbrooke (Québec), QC J1M, Canada; patrick.bergeron@ubishops.ca; 4 Institute National de Santé Publique du Québec (INSPQ), 945 Wolf Avenue, Québec (Québec), QC G1V 5B3, Canada; pierre.gosselin@inspq.qc.ca (P.G.); claudia.blais@inspq.qc.ca (C.B.); louis.rochette@inspq.qc.ca (L.R.); diane.belanger@crchudequebec.ulaval.ca (D.B.); 5 Research center of the Centre Hospitalier Universitaire de Québec (CHUQ), Delta II building, 6th floor, 2875 Laurier Boulevard, Québec (Québec), QC G1V 2M2, Canada; 6 Faculty of Pharmacy, Université Laval, Pavillon Ferdinand-Vandry, 1050 Avenue de la Médecine, Québec (Québec), QC G1V 0A6, Canada; 7 The Eau Terre Environment Research center, Institute National de la Recherche Scientifique (INRS), 490 Couronne Street, Québec (Québec), QC G1K 9A9, Canada; fateh.chebana@ete.inrs.ca

**Keywords:** climate change, floods, public health, health problems, cardiovascular diseases, Canada

## Abstract

*Background:* Floods represent a serious threat to human health beyond the immediate risk of drowning. There is few data on the potential link between floods and direct consequences on health such as on cardiovascular health. This study aimed to explore the impact of one of the worst floods in the history of Quebec, Canada on acute cardiovascular diseases (CVD). *Methods:* A cohort study with a time series design with multiple control groups was built with the adult population identified in the Quebec Integrated Chronic Disease Surveillance System. A geographic information system approach was used to define the study areas. Logistic regressions were performed to compare the occurrence of CVD between groups. *Results:* The results showed a 25%–27% increase in the odds in the flooded population in spring 2011 when compared with the population in the same area in springs 2010 and 2012. Besides, an increase up to 69% was observed in individuals with a medical history of CVD. *Conclusion:* Despite interesting results, the association was not statistically significant. A possible explanation to this result can be that the population affected by the flood was probably too small to provide the statistical power to answer the question, and leaves open a substantial possibility for a real and large effect.

## 1. Introduction

The Intergovernmental Panel on Climate Change predicts that climate change is likely to cause an increase in flood hazards in many areas of the world [1]. Floods are the most frequent natural disasters in Canada, occurring almost five times as often as the next most common disaster, wildfire [2]. There is also a strong consensus on the increase in the intensity and frequency of precipitation in the province of Quebec, Canada which is likely to cause more flooding in the coming decades [3].

Floods represent a serious threat to human health beyond the immediate risk of drowning. They can increase exposure to toxins and pathogens may have implications for mental health, and can disrupt the capacity of health care systems to respond to health crises [4,5,6,7]. These environmental disasters are also known to be associated with intense stress and the individuals involved in such events are often forced to make unusual efforts [8,9]. However, there is little data on the potential link between floods and direct consequences on health, such as cardiovascular diseases (CVD). In fact, a few studies have reported an association between natural disasters on the increased occurrence of CVD, such as after the tsunami in Japan in 2011 [10]. This study reports a significant increase in acute heart failure in the tsunami area when compared to the predisaster period. The maximal peak was observed three to four weeks after the event [10]. Since CVD is the second most important causes of death in Canada [11], a better understanding of the impact of these weather disasters on cardiovascular health will allow us to improve the surveillance and the prevention of these diseases as well as providing empirical data to elaborate strategic action plans on patient care and health services. Furthermore, climate change increases the need for research, both for the assessment of future health burdens and improving analysis of current and future options for health-related response [7].

**Figure 1:**
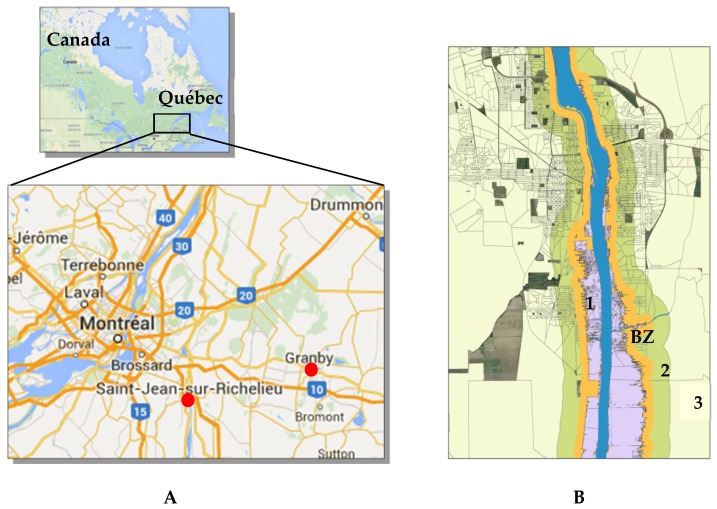
(**A**) Map of the province of Quebec in Canada, as well as the cities of Saint-Jean-sur-Richelieu and Granby; (**B**) Map of the studied areas in Saint-Jean-sur-Richelieu (1: Flooded area; 2 and 3: Control areas; BZ: Buffer zone (200 meters)).

Spring snowmelt accompanied by rainfall is the major cause of flooding in the province of Quebec, Canada [12]. This was the case of the flood in the city of Saint-Jean-sur-Richelieu in 2011 (located near Montreal, Figure 1A). It was the longest and one of the worst floods in the recent history of Quebec [13]. From 22 April to 14 June 2011, the rapid snowmelt conjugated with torrential rain falls that lasted for several weeks, led to increasing water levels of Lake Champlain and the Richelieu River to critical thresholds never registered before, causing a major overflow in Saint-Jean-sur-Richelieu [14]. It took nearly four months until the complete resorption of the water in the city. During this period, residents of 1619 homes were evacuated and overall, 2663 homes were flooded to various extents [15]. The purpose of this study was to evaluate the effects of this flood on the occurrence of acute CVD. This study was part of a ministerial program aimed at a better understanding of the association between cardiovascular diseases and climate change in Quebec.

## 2. Methods

### 2.1. Design

To measure the relationship between the flood in Saint-Jean-sur-Richelieu in spring 2011 and the occurrence of acute CVD, a population-based retrospective cohort study was conducted using a time series design with multiple control groups.

#### Defining the Study Areas Using a Geographic Information System (GIS) Approach

A GIS approach was used to define the flooded area (area 1) and two control areas in the same town (areas 2 and 3) according to the postal codes (Figure 1B). The flooded population and the non-flooded population were separated by a defined buffer zone of 200 meters to insure the absence of flooded postal codes in the control area (Figure 1B). A town nearby Saint-Jean-sur-Richelieu (Granby, Figure 1A) with a similar population size, a comparable socioeconomic status and the presence of a river not affected by the flood in spring 2011, was also selected as a control population (area 4).

**Figure 2:**
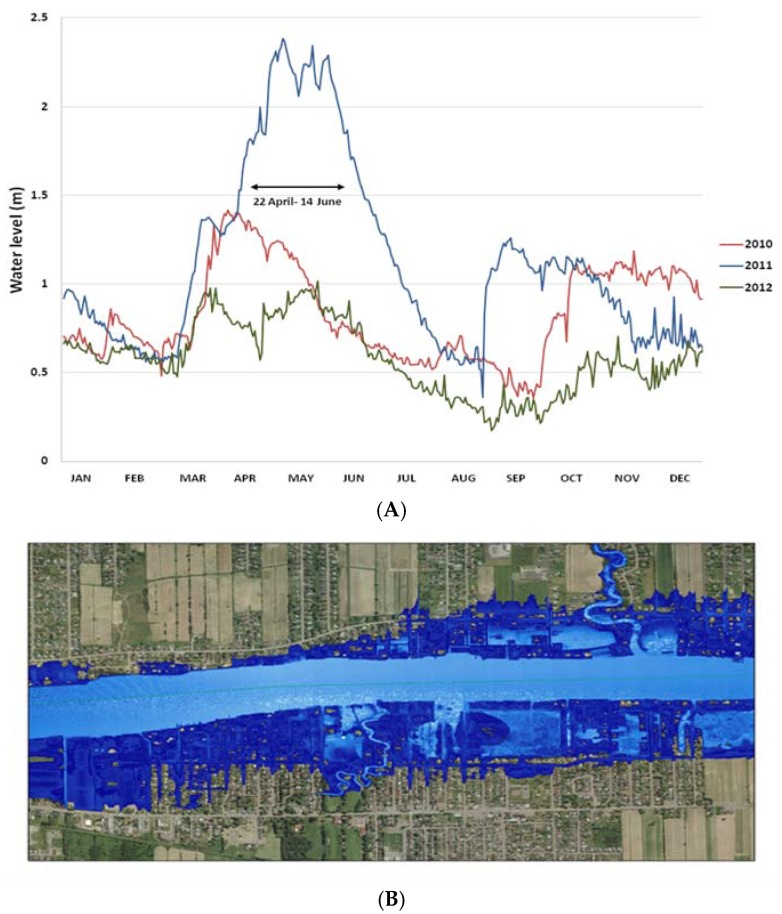
Geographic Information System (GIS) approach: defining the study areas. (**A**) Water levels of the Richelieu River in 2011 compared to 2010 and 2012; (**B**) Identification of the areas affected by the flood using the ArcGIS software.

GIS technology has become a powerful tool to map the extent of such events and investigate public health issues [16]. The GIS component of the study was addressed using data on water levels of the Richelieu River and topographic elevation maps. Water levels were obtained from the hydraulic records of the Government of Canada who maintains a hydraulic measuring station in Saint-Jean-sur-Richelieu (station 02OJ016) (Figure 2A). Data were available through the National Water Data Archive (HYDAT database) [17], which provides information on the water flow, daily values on water levels in real time and archived since 1972 [18]. Topographic maps were obtained from the airborne remote sensing LiDAR (Light Detection and Ranging) technology that provides very accurate maps of the area. Data on water levels were paired to the high resolution topographic LiDAR maps for the year of 2011, using Arc Hydro tools in the ArcGIS software (version 10.2) (Environmental Systems Research Institute Inc.: California, CA, USA) (Figure 2B). The number and extent of postal codes affected by the flood were extracted to identify the study cohorts (presented in Figure 1B). The flooded population (area 1) consists of 271 postal codes flooded to various extents, 119 of which had more than half of their surface area flooded. The control area 2 near the river was delimited by a 500-meters wide zone and included 1003 postal codes while the control area 3 included 1314 postal codes. These control areas were defined as such in order to provide a balanced coverage of the area.

### 2.2. Health Data Sources

This study used medico-administrative data extracted from the Quebec Integrated Chronic Disease Surveillance System (QICDSS) created by the *Institute* *National de* *Santé* *Publique du Québec* (INSPQ) [19]. The QICDSS is an innovative chronic disease surveillance system developed in order to monitor several chronic diseases in Quebec, such as cardiovascular diseases. The QICDSS data are derived from the linkage of five health administrative databases administered by the provincial health insurance board (*Régie de l’Assurance* *Maladie du Québec* *(RAMQ))*. The QICDSS contains information on all individuals covered by the Quebec universal public health insurance plan that had or are at risk of at least one of the studied chronic diseases. The five data sources are the health insurance registry, the hospitalization database, the physician claims database (including emergency department (ED) visits, outpatient visits and community physicians’ visits), the pharmaceutical services database (for people aged 65 years and older), and the vital statistics death database. The health insurance registry contains information on patient demography (age, gender), geographic localization, as well as the eligibility to the province’s public health and drug insurance plans. The hospitalization database provides information on inpatient discharges from Quebec hospitals. The data are related to the hospital stay (e.g., date, location, and duration), diagnoses (e.g., primary and secondary diagnoses), services, intensive care and interventions. Diagnostic codes are based on the international statistical classification of diseases and related health problems, 9th Revision (ICD-9) up to 31 March 2006, and the Canadian enhancement of the 10th Revision (ICD-10-CA) thereafter. The physician claims database provides data related to fee-for-service billing. These include the diagnosis code associated with the service rendered (ICD-9 coding system), the treating physician and the location where the service was provided. Using a unique encrypted identifier, patient data with specific selected chronic diseases from all these databases were merged to create the QICDSS.

### 2.3. Study Population

The adult populations (≥20 years old) of the different study areas were identified according to the postal code of their residence in the QICDSS database. To be part of the cohorts, subjects had to be covered by the Quebec public health insurance plan at least one day in the fiscal year in which acute CVD is assessed. About 99% of the adult population in Quebec is covered by the public health insurance plan [20].

### 2.4. Cardiovascular Outcomes

An acute cardiovascular event was defined as a diagnosis of a major or a potentially fatal CVD associated with an ED visit or with a hospitalization (main cause and urgent). All diagnoses were identified using the ICD-9 and ICD-10-CA codes [21]. Precisely, CVD assessed were coronary diseases (ICD-9 codes: 410-414 and ICD-10-CA codes: I20-I24) and heart failure (ICD-9 code: 428 and ICD-10-CA code: I50).

The occurrence of acute CVD was compared between the flooded population of area 1 and the non-flooded populations of areas 2, 3 and 4 during a 4-month period, from 1 April 2011, to 31 July 2011 (spring 2011). The occurrence of acute CVD was also compared at three different periods in time in the affected area (area 1). Thereby, the period corresponding to the flood (spring 2011) was compared to the same 4-month period of spring 2010 (1 April 2010, to 31 July 2010) and of spring 2012 (1 April 2012, to 31 July 2012).

### 2.5. Confounders

Age, gender, Charlson comorbidity index (adapted for the Quebec health administrative databases by D’Hoore *et al.* [22]), history of CVD, most common risk factors for CVD (hypertension and diabetes) and mental health disorders were included as potential confounders of the association between the flood and the occurrence of acute cardiovascular events. Since the history of CVD and diabetes were included as separated cofounders, these conditions were excluded from the Charlson Comorbidity index calculation. History of CVD [23,24,25] was defined as a hospitalization with a main or a secondary diagnosis of CVD (ICD-9 codes: 410–414, 428, 362.3, 430–432, 434–436 and ICD-10-CA codes: I20–I25, I50, G45 (excluding G45.4), H34.0, H34.1, I60, I61, I63 ((excluding I63.6), I64) or a coronary intervention (The Canadian Classification of Diagnostic, Therapeutic and Surgical Procedures (CCP) codes: 48.02, 48.03, 48.11–48.19 and The Canadian Classification of Health Interventions (CCI) codes: 1.IJ.50, 1.IJ.57.GQ, 1.IJ.54, 1.IJ.76) or at least two ambulatory visits for CVD in a one-year span between 1996 and the beginning of the study period. History of hypertension [26] was defined as a hospitalization with a main or a secondary diagnosis of hypertension (ICD-9 codes: 401–405 and ICD-10-CA codes: I10–I13, I15) or at least two ambulatory visits for hypertension in a two-year span between 1996 and the beginning of the study period. History of diabetes [27] had the same definition as hypertension with their corresponding ICD-9 and ICD-10-CA codes: 250 and E10–E14, respectively. Finally, history of mental disorders was defined as a hospitalization with a main or a secondary diagnosis of mental disorders (ICD-9 codes: 290–319 and ICD-10-CA codes: F00–F99) or at least one ambulatory visits for a mental disorder in the year before the beginning of the study period.

### 2.6. Statistical Analysis

Chi-square tests were conducted to examine differences in the occurrence of acute cardiovascular events according to the area (area 1 *vs.* areas 2, 3 or 4), and the period (spring 2011 *vs.* spring 2010 or 2012). A multivariate logistic regression with repeated measures using generalized estimating equations (GEE) was done to compare the risk of acute cardiovascular events between the flooded population (area 1) in spring 2011 and the population in the same area in spring 2010 and in spring 2012. This analysis was repeated in the sub-population who had a history of CVD in order to measure the impact of the flood within this most vulnerable population. In these cases, a GEE method is used in place of basic regression approaches because the population in the same area was evaluated over time and the study variables were correlated, thus violating independence assumptions made by traditional regression procedures [28]. To compare the risk of acute cardiovascular events between the flooded population (area 1) and the control populations (areas 2, 3 and 4) during the period corresponding to the flood (spring 2011), a traditional multivariate logistic regression was applied. Models were adjusted for the cofounders mentioned above and adjusted odds ratios (OR) with 95% confidence intervals (CI) were presented. All analyses were performed using SAS 9.2 Statistical Software (SAS Institute Inc.: Cary, NC). All *p*-values were two-sided, and *p*-values of ˂0.05 were considered to be statistically significant. 

### 2.7. Ethical Aspects

The processes of creating the QICDSS and data access both meet stringent standards of security and privacy. Government bodies in legal possession of the databases, the public health ethics committee and the *Commission d’accès à l’information du Québec* evaluated and approved the creation process and the surveillance that can be done with this system. The study was conducted according to an agreement established between the *INSPQ* and the government of Quebec in part of the ministerial plan of multithematic surveillance. This plan has received its approval by the Public Health Ethic Committee in January 2010 (ISBN: 978-2-550-58576-3) [29].

## 3. Results

In spring 2011, 10,081 adults covered by the public health insurance plan had their primary residence in the flooded area of Saint-Jean-sur-Richelieu. Patient characteristics of the different study areas are presented in Table 1. When compared with the control populations (areas 2, 3, and 4), male/female ratio is slightly higher in the flooded population. There are also lower proportions of individuals with a high comorbidity index (≥2), as well as with medical history of mental disorders, hypertension, diabetes and CVD within this population when compared to control populations of areas 2 and 4. However, these characteristics seem similar between the flooded population and the control population of area 3 (one of the control areas located in Saint-Jean-sur-Richelieu). Baseline characteristics of individuals in the flooded area were relatively stable throughout the years of 2010 to 2012 (data not shown).

**Table 1:** Baseline characteristics of the flooded and the control populations in spring 2011.

Characteristics	Flooded Area	Control Areas			*p*-Value
	**Area 1**	**Area 2**	**Area 3**	**Area 4**	
*n*	10,081	22,654	40,119	38,463	
Male sex, *n* (%)	5168 (51.3%)	10,874 (48.0%)	19,402 (48.4%)	18,270 (47.5%)	<0.001
Age (year), mean ± SD	49.6 ± 16.6	49.1 ± 17.7	48.5 ± 17.3	51.7 ± 18.3	<0.001
Comorbidity score, *n* (%)					
0	9154 (90.8%)	20,325 (89.7%)	36,377 (90.7%)	33,844 (88.0%)	<0.001
1	760 (7.5%)	1843 (8.1%)	2995 (7.4%)	3625 (9.4%)	
≥2	167 (1.7%)	486 (2.2%)	747 (1.9%)	994 (2.6%)	
Mental disorders, *n* (%)	548 (5.4%)	1546 (6.8%)	2334 (5.8%)	2243 (5.8%)	<0.001
Diabetes, *n* (%)	790 (7.8%)	2041 (9.0%)	3221 (8.0%)	3792 (9.9%)	<0.001
Hypertension, *n* (%)	2328 (23.1%)	5475 (24.2%)	9282 (23.1%)	9989 (26.0%)	<0.001
History of CVD, *n* (%)	1021 (10.1%)	2620 (11.6%)	4170 (10.4%)	4906 (12.8%)	<0.001

CVD: cardiovascular diseases, *n*: number, SD: standard deviation.

### 3.1. Flooded Area in Spring 2011 vs. Spring 2010 and Spring 2012

**Figure 3:**
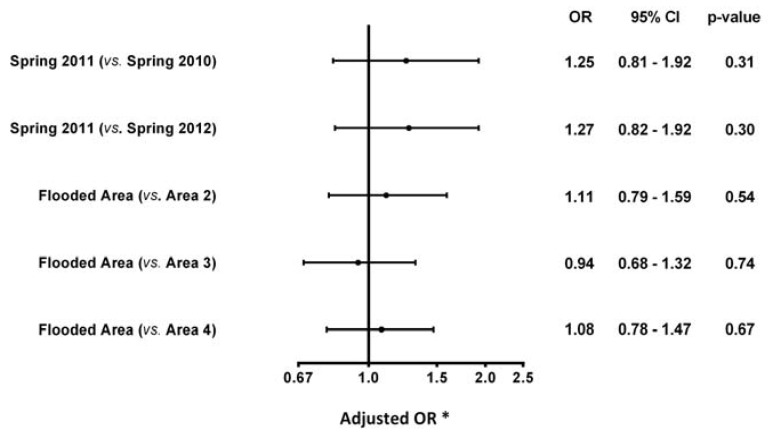
Risk of acute CVD according to different periods of time and areas. * Adjusted for age, gender, Charlson comorbidity index, history of CVD, diabetes, hypertension, and mental health disorders. CI: confidence intervals, OR: odds ratio.

**Figure 4:**
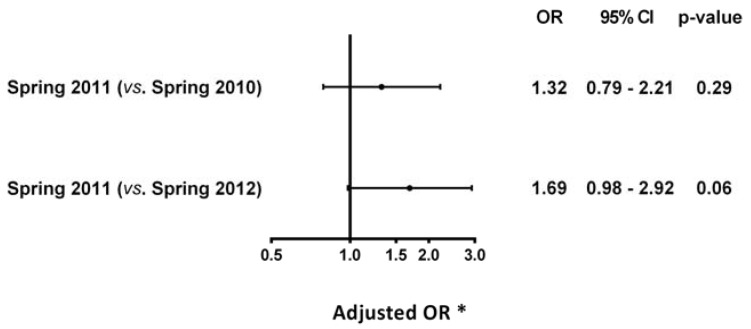
Risk of acute CVD among individuals with a medical history of CVD. * Adjusted for age, gender, Charlson comorbidity index, diabetes, hypertension, and mental health disorders. CI: confidence intervals, OR: odds ratio.

In the flooded area, the rate of acute CVD during spring 2011 was 0.47%, which corresponded to 47 events reported in an ED visit or reported as the main diagnosis of a hospitalization (Table 2). A higher incidence of acute cardiovascular events was observed during this period compared with the same 4-month period of the previous year (spring 2010: 0.37%) and the year after (spring 2012: 0.38%) (Table 2). However, as shown in Figure 3, this increase was not statistically significant according to the logistic regression analysis, which controlled for patient-level cofounders (spring 2011 *vs.* spring 2010 OR 1.25, 95% CI 0.81 to 1.92; spring 2011 *vs*. spring 2012 OR 1.27, 95% CI 0.82 to 1.92). The difference in the rate of acute CVD observed in spring 2011 when compared to spring 2012 increased noticeably when the analyses were performed in a sub-population with a history of CVD and was almost statistically significant (OR 1.69, 95% CI 0.98 to 2.92) (Figure 4).

**Table 2:** Occurrence of acute CVD during springs 2010 to 2012, and according to the different study areas.

Areas	Spring 2010; *n* (%)	Spring 2011; *n* (%)	Spring 2012; *n* (%)
Flooded Area	37/10,006 (0.37%)	47/10,081 (0.47%)	38/10,128 (0.38%)
Area 2	122/22,456 (0.54%)	113/22,654 (0.50%)	121/22,789 (0.53%)
Area 3	220/39,807 (0.55%)	208/40,119 (0.52%)	193/40,408 (0.48%)
Area 4	207/38,162 (0.54%)	230/38,463 (0.60%)	234/38,517 (0.61%)

CVD: cardiovascular diseases. *n*: number. SJSR: Saint-Jean-sur-Richelieu.

### 3.2. Flooded Area (Area 1) vs. Control Areas (Areas 2, 3 and 4) in Spring 2011

As seen in Table 2, a lower rate of acute cardiovascular events was observed in the flooded area (0.47%) when compared with the control areas (0.50%, 0.52%, and 0.60%, respectively) in spring 2011. However, as previously mentioned, the population in the flooded area had lower proportions of individuals with a high comorbidity index, and with a medical history of diabetes, hypertension, CVD, as well as mental disorders. In fact, in the adjusted model, the flooded area was positively (but not statistically) associated with a slight increase in acute CVD during spring 2011 when compared with control areas 2 and 4 (Area 1 *vs.* Area 2 OR 1.11, 95% CI 0.79 to 1.59; Area 1 *vs*. Area 4 OR 1.08, 95% CI 0.78 to 1.47) over the same period (Figure 3). 

## 4. Discussion

The present study did not detect a significant association between the flood of Saint-Jean-sur-Richelieu in spring 2011 and the occurrence of acute CVD when controlling for patient-level cofounders. While the principal p-values were far from significant, the estimated effect sizes were large: an increase in the odds of 25%–27% in the flooded area when compared with the same area the previous year and the year after, and an increase of up to 69% when the analysis was restricted to individuals with a history of CVD. This suggests that the population affected by the flood was too small to provide the statistical power to answer the question, and leaves open a substantial possibility for a real and large effect.

Previous studies have reported a positive relationship between natural disasters and the occurrence of CVD [10,30,31]. Nakamura *et al*. [10] compared the appearance of acute heart failures between the area devastated by the tsunami in Japan in 2011 and a control area. Using a ratio that represented the degree and extent of damage caused by the tsunami in the residential area of each town (percent tsunami flood area per built-up area) to define the study groups, the authors reported a significant increase in acute heart failures for several weeks in the area devastated by the tsunami (maximum peak observed three to four weeks following the disaster). Moreover, they observed that this growth was significantly correlated with the degree of tsunami-induced destruction in residential areas or with the number of evacuees [10]. Aoki *et al*. [31] also observed that the weekly occurrences of CVD, including heart failure, acute coronary syndrome, stroke and cardiac pulmonary arrest were all significantly increased after the Great East Japan Earthquake in 2011 when compared with the previous three years, independently of age, gender, or residence area. Monitoring data of the hurricane Katrina, which aimed to evaluate the long-term incidence of acute myocardial infarctions in the 3 years following the storm, showed an important cardiovascular mortality and increased medical consultations related to CVD when compared with the two-year period before the event [30]. However, the post-Katrina group had a greater prevalence of unemployment, lack of medical insurance, smokers, medical noncompliance, and first-time hospitalizations, history of coronary artery disease, multiple vessel disease and percutaneous coronary intervention (all *p* < 0.05). The evaluation between groups was not adjusted for these confounders [30].

Many hypotheses may explain the lack of significant effect between the flood and acute cardiovascular events in the present study. First of all, the flood of Saint-Jean-sur-Richelieu was less severe and damaging than the ones reported in the literature so far. Indeed, the tsunami in Japan in 2011 caused thousands of deaths and major material damages [10]. Secondly, the limited extent of the flood of Saint-Jean-sur-Richelieu and the emergency measures in place may have contributed to the fast delocalization of people and the quick management and support of those in need, unlike other flood disasters. In Saint-Jean-sur-Richelieu, disaster victims received housing and food [14]. Additionally, access to health care services was not affected: ambulatory clinics, hospitals and pharmacies were located outside the flooded area. Those factors could have had a positive impact on the prevention of urgent care utilization. In previous studies, one of the hypotheses of the increase of acute CVD was based on the failure in the continuum of health care during the disaster. A study conducted in Japan in 2006 [32] reported a higher prevalence of interruption of medication among subjects in a flooded area that had to be evacuated compared to individuals not affected by the flood (9% *vs*. 23%), and those who experienced interruption of medication were more likely to have deteriorated their health status one month after the event (OR 4.5, 95% CI 1.2–17.6) [32]. Third, unlike other flood disasters, such as the tsunami in Japan, the rising of water levels in Saint-Jean-sur-Richelieu was slower and progressive. Thus, people affected by the flood could have had more time to be prepared for the event. Finally, it is known that loss of human life and material damage associated with environmental disasters might have major psychological consequences [33]. Many studies have reported a state of post-traumatic stress after major disasters in the individuals concerned [6,34,35,36,37]. The intense stress induced during these events may have significant consequences on health, such as cardiovascular health. Some evidences suggest that acute stress may be related to adverse cardiac outcomes particularly in individuals at risk or with history of CVD through the activation of the sympathetic nervous system [38,39,40]. In fact, it may lead, among others, to the elevation of the heart rate and the blood pressure as well as a transient increase in blood viscosity. Kershaw *et al*. [41] have demonstrated that highly stressful life events were associated with a higher incidence of CVD independently of sociodemographic factors and depressive symptoms. However, in the case of Saint-Jean-sur-Richelieu, the stress-induced in individuals affected by the flood may have been attenuated by the absence of deaths (as reported by the media and authorities) as well as the rapid delocalization and support of the victims. Overall, 7000 psychosocial interventions were made during the period of the disaster [14]. All these factors may have resulted in fewer impacts on the physical and emotional plan of the victims.

## 5. Strengths and Limitations

The major strength of this study is the use of a GIS approach based on hydraulic and topographic data in order to extrapolate the extent of the flood. This method is a powerful tool that can easily be transposed to other events to investigate public health issues. Moreover, the use of data from provincial databases permitted accurate assessment of the occurrence of acute CVD among the studied populations.

Several limitations should be mentioned. First, it was not possible to evaluate the cardiovascular mortality associated with the flood because this information was not available at the time of the study. Second, it is possible that some individuals who had a residence in Saint-Jean-sur-Richelieu, were not at home during the flood period. However, every adult covered by the public health insurance who had a residence in the flooded area during this period was considered as the case-population. This may have resulted in an underestimation of the incidence of acute cardiovascular events for the population living in the flooded area. Finally, the lack of statistical power did not allow us to clearly establish an association between the flood and the occurrence of acute CVD.

## 6. Conclusions

In conclusion, there were about 25% to 27% more cases of acute cardiovascular events during the flood period (spring 2011) at Saint-Jean-sur-Richelieu as compared with the same period in 2010 and 2012. This increase was even more pronounced in individuals with a medical history of CVD (up to 69% more cases of acute CVD within this population). However, the impact of the flood of Saint-Jean-sur-Richelieu on acute CVD could not be established. It may be explained by several factors: (1) the relatively minor impact and extent of the disaster; (2) the access to health care services was not affected by the flood having minor impacts on the continuum of healthcare during the disaster; and (3) the efficacy and the rapidity of the management at a regional and at a provincial level. Further studies, based on a similar methodology but with larger sample sizes, may be needed to provide more robust evidences. 
